# Board scores in the spotlight: Public reporting and the unintended consequences

**DOI:** 10.1002/aet2.70006

**Published:** 2025-03-31

**Authors:** Matthew E Kelleher, Sally A Santen, Christiana Draper, Jaime Jordan, Michael Gottlieb, Benjamin Kinnear

**Affiliations:** ^1^ Department of Pediatrics University of Cincinnati College of Medicine/Cincinnati Children's Hospital Medical Center Cincinnati Ohio USA; ^2^ University of Cincinnati College of Medicine Cincinnati Ohio USA; ^3^ Virginia Commonwealth University Medical Center Richmond Virginia USA; ^4^ Oregon Health & Science University Portland Oregon USA; ^5^ Department of Emergency Medicine Rush University Medical Center Chicago Illinois USA

The American Board of Emergency Medicine (ABEM) recently announced plans to publicly report program‐level board certification examination pass rates.^1^ This initiative will present program‐level board pass rates for public viewing. Multiple arguments can be made for such a change. Medical education is increasingly seen as a service for which trainees have paid large sums of money and sacrifice a significant amount of time and effort. Transparency and accountability to learners that show services are high quality is important. Additionally, public reporting of program board pass rates provides a mechanism for accountability to society at large and aligns with ABEM's mission “To ensure the highest standards in the specialty of Emergency Medicine.”^2^ Graduate medical education (GME) is largely funded through tax dollars, and an argument can be made that the public should be able to see the degree to which different training programs are helping their graduates pass certifying examinations.^3^ Finally, this change would align ABEM with multiple other major American Board of Medical Specialties (ABMS) member boards who publicly report program pass rates, such as the American Board of Internal Medicine, American Board of Pediatrics, and American Board of Family Medicine.^4^ These rationales have merit, but unintended consequences lurk around the corner. In this perspective, we describe the potential negative impact of publicly reporting program‐level certifying examination pass rates. Specifically, we explore how public reporting could disincentivize holistic review of applicants during residency recruitment. We propose actionable strategies that various stakeholders may consider for balancing transparency with the broader mission of holistic review and inclusive recruitment practices in GME.

## BEWARE OF THE COBRA EFFECT

Humans are influenced by incentive structures. As rational actors, we tend to (either implicitly or explicitly) alter our thinking and behavior when different incentive structures are presented to us. When such structures lead to unwanted consequences, they are labeled “perverse incentives.” A colloquial term for the impact of a perverse incentive is sometimes called the *Cobra Effect*.^5^ The Cobra Effect draws from an anecdote in which a governmental effort to reduce the number of cobras in Delhi, India, backfired. The initiative offered a bounty for dead cobras, so people began breeding cobras to turn in more dead snakes. The bounty was intended to incentivize the killing of cobras, hence decreasing the overall population. However, people quickly realized they could game the system for financial gain, leading to an overall increase in the cobra population. The incentive structure led to unwanted consequences that could potentially have been predicted by considering how people would respond.

We believe that publicly reporting program certifying examination pass rates may lead to its own Cobra Effect, potentially altering program incentives in ways that undermine holistic recruitment efforts. Holistic review in residency selection has gradually shifted programs away from an overreliance on academic metrics like test scores and class rank in favor of a broader (i.e., more holistic) evaluation of an applicant's strengths, interests, and value alignment with a residency.^6^ While academic metrics still play a role, they are no longer the sole focus, thanks to growing awareness of their limitations. Many traditional academic metrics lack validity evidence for use in GME recruitment and do not predict future residency performance.^7^, ^8^, ^9^ Additionally, concerns about inequity and bias in these metrics—such as the systematic disadvantages faced by minoritized groups—have highlighted the need for more inclusive strategies.^10^, ^11^ Holistic review, while not a standardized approach, is recommended by most national organizations (AAMC, AMA, ACGME) because of its potential to improve equity in the residency selection process.^12^, ^13^, ^14^, ^15^, ^16^ The competency‐based medical education (CBME) movement has further underscored that knowledge and test‐taking ability are only parts of a broader framework of competence.^17^ We fear that publicly reporting program board pass rates for the sake of transparency and accountability may underestimate the looming Cobra Effect of incentivizing programs to return to *overemphasizing* test scores when evaluating applicants.

## STANDARDIZED TEST SCORES PREDICT FUTURE STANDARDIZED TEST SCORES

To be clear, we are not suggesting that passing the certifying examination is unimportant. When compared to individuals who are not board certified, board certification has been linked to meaningful patient care outcomes in future practice.^18^, ^19^, ^20^, ^21^ Thus, the certifying examination pass rate presents a metric that has some validity evidence to support its use in assessing *individual* graduates’ readiness for practice. The key issue lies in differentiating whether the residency program's quality or the individual's test‐taking abilities has more influence on passing or failing the examination. The rationale for sharing program pass rates relies on a false premise: that all programs recruit learners who enter residency with a similar chance of passing the certifying examination. However, it is very unlikely that testing ability and medical knowledge is equally among matriculating cohorts across programs. The best predictor of passing a standardized certifying examination is performance on previous standardized examinations.^22^, ^23^, ^24^, ^25^, ^26^, ^27^, ^28^, ^29^, ^30^, ^31^ In contrast, the evidence is much less clear about whether the quality of residency training is associated with board passage rates.^32^, ^33^, ^34^, ^35^, ^36^, ^37^, ^38^, ^39^, ^40^ Use of program‐level board pass metrics as a signal of residency training quality fails to account for inherent differences in programs’ incoming residents' *prior* standardized examination performance, making intraprogram comparisons potentially misleading.

## PUBLIC REPORTING OF BOARD SCORES: NEW INCENTIVE STRUCTURES

The public reporting of board certification pass rates will likely exert significant pressure on program directors (PDs) to mitigate the risk of examination failures. This is a good goal to aspire toward; PDs should help their graduates pass the certifying examination. However, they may feel pressured to avoid failures not only by improving education and training, but also by changing recruiting practices. Even a small number of individual examination failures could greatly alter a program's overall pass rates given the relatively low number of graduates per year from ABEM training programs. Programs might fear risk of impaired recruiting should applicants use program board pass rates as a metric to evaluate and rank programs. The most straightforward way for PDs to minimize risk is to recruit applicants with the least risk of examination failure. This aligns with Campbell's Law, which suggests that when any measure is emphasized as a marker of social standing (e.g., high pass rates equates to a better residency training program), it will be prone to distortion or corruption.^41^ In this context, program board pass rates taking on such a visible and elevated status will no doubt make prior testing performance a more important metric for PDs to consider when making decisions on whom to invite for an interview and how to rank them on final lists. PDs, as rational actors, may make decisions to “protect” their programs as well as their personal reputation by prioritizing metrics that maximize pass rates and minimize examination failures. If programs focus significantly on recruiting applicants with proven test‐taking abilities, the entire system shifts toward this strategy. In turn, this could lead to students and schools responding by placing even more emphasis on testing metrics. If a residency program chooses to resist this shift on principle, maintaining a more holistic review, they are opening themselves up to scrutiny from the public and applicants if their pass rates begin to lag behind other programs. Ultimately, a cycle of competitive defensiveness is perpetuated over time.

Public reporting of program board pass rates risks undermining national momentum made toward programs using holistic review for applicant selection. For many programs, the added pressure of transparency may act as a disincentive to invest in resource‐intensive, nuanced holistic review processes. Instead, it may nudge programs—if not outright shove them—back toward relying heavily on easily sortable academic metrics like test scores. Programs that are already stretched thin and searching for simpler application review strategies may find this shift particularly appealing. Holistic review has led to an emphasis on increasing the number of diverse residents in programs nationwide. A return to prior selection strategies has potential to undermine the diverse representation of physicians, which we know has a positive effect on patient outcomes.^42^, ^43^, ^44^ Public reporting could inadvertently set back holistic review efforts, prioritizing expediency over the broader, equity‐driven goals that have shaped medical education reform in recent years.

## OUR APPEALS TO STAKEHOLDERS

### To the ABEM

As ABEM considers publicly reporting program‐level board certification pass rates, it is imperative to weigh the potential unintended consequences against the intended benefits. Transparency and accountability are important virtues, but the resulting Cobra Effect among programs could erode the very goals this policy seeks to achieve. Have all relevant stakeholders been engaged? Do learners desire this transparency? If so, would their opinions change if they understood how public reporting might incentivize programs to deprioritize holistic review in favor of test metrics‐based selection strategies?

We suggest three specific considerations. (1) Consider using an adjusted program pass rate that can account for the baseline likelihood of passing for each individual resident and thus residency program cohorts. There are numerous data sources that could provide context to a residency program's pass rate.^45^ While the ultimate goal is to help every graduate pass the certifying examination, in reality a program's pass rate is dependent on much more than the quality of training it provides. (2) Consider reporting pass rates categorically—such as above or below a rational threshold that has been shown to be a meaningful target for residency programs. For example, one study has shown that graduates from residency programs with a >80% pass rate had lower in‐hospital mortality and length of stay.^18^ Reporting based on a criterion would significantly help in reducing the risk of overinterpreting small differences between programs (e.g., 95% vs. 91% pass rate) that effectively should be interpreted as having no meaningful difference. If most programs have 12 graduates, the confidence intervals and standard error around any difference is likely to be misinterpreted. (3) Consider how program pass rates will be collated and reported. Single‐year pass rate data can swing widely based on a single individual's examination failure, making this method of reporting especially susceptible to misinterpretation and misuse. Reporting a rolling average over at least 3 years would help to mitigate these risks and be more in line with other ABMS specialty boards. (4) Share detailed individual score reports with the PDs to drive programmatic improvement and curricular gap analysis.

### To applicants

As prospective residents, consider carefully what a program's board pass rate signifies and what it does not. While a low pass rate might suggest an educational curriculum that poorly prepares residents for the certifying examination, it could just as easily reflect a program's willingness to recruit and select learners who are not strong test takers but are high‐quality physicians. Differences in program pass rates may also represent statistical noise or construct‐irrelevant variance rather than meaningful distinctions in program quality. For example, if a program has 12 graduates, one failure versus two failures will be an almost 10% difference in pass rates (11/12 pass is 92%, 10/12 is 83%). A quick glance at these differences posted online may feel significant, but there is risk in drawing any meaningful conclusion. Just like comparing applicants based only on a select metric could lead to faulty conclusions, so could comparing programs based on similar metrics. The culture and support that a program provides to its residents may be more important than their board passage rates. Resist the temptation to reduce a program's quality to measurable metrics. Instead, investigate how a program helps residents develop into competent independent physicians. Read their website, talk to current residents, and ask probing questions during recruitment. Remember that many factors contribute to an individual passing a certifying examination—personal circumstances, program resources, and random chance all play a role. A single number is unlikely to capture a program's quality or its alignment with your goals.

### To PDs

Resist the temptation to bend to Campbell's Law. As educators, our mission is to nurture and develop future physicians, not to game metrics. The word “educate” comes from the Latin *ēducō*—to bring up, rear, or train. If our primary focus is recruiting and graduating great test takers, what have we truly accomplished as educators? Instead, focus on recruiting individuals who align with your program's mission, serve diverse patient communities, and demonstrate the potential to grow into outstanding physicians. This will require courage, as the public reporting of pass rates may negatively reflect upon you if your program's numbers falter. Stand firm in your commitment to holistic review, identifying and nurturing good humans who will be good physicians, not just good testers—a distinction that need not be mutually exclusive. Advocate for your approach, both in recruitment discussions and in securing institutional support. Your decisions today shape the future of medical education and patient care.

## CONCLUSION

Medical education has made great strides toward holistic recruitment strategies. The potential unintended consequences highlighted by Campbell's Law and the Cobra Effect reveal the risk of incentivizing programs to return to an overemphasis on test‐taking abilities in resident selection. More research is needed to explore the complex alchemy of how certifying examination results can be transparently reported for stakeholders without disincentivizing holistic recruitment and selection practices. While we encourage residency programs to share program board pass rates with prospective applicants during the recruitment process when there is an opportunity to provide appropriate context and nuanced discussion, we recommend that ABEM not make board pass rates public.

## CONFLICT OF INTEREST STATEMENT

The authors declare no conflicts of interest.

## Data Availability

Data sharing not applicable to this article as no datasets were generated or analysed during the current study.
